# Efficacy and safety of gyejigachulbutang (Gui-Zhi-Jia-Shu-Fu-Tang, Keishikajutsubuto, TJ-18) for knee pain in patients with degenerative knee osteoarthritis: a randomized, placebo-controlled, patient and assessor blinded clinical trial

**DOI:** 10.1186/s13063-019-3234-6

**Published:** 2019-02-19

**Authors:** Jae-Uk Sul, Myung Kwan Kim, Jungtae Leem, Hee-Geun Jo, Sang-hoon Yoon, Jeeyong Kim, Eun-Jung Lee, Jeong-Eun Yoo, So Jung Park, Young Il Kim, Eunseok Kim, In Chul Jung, Ju-Hyun Jeon, Yang-Chun Park

**Affiliations:** 1 Chung-Yeon Central Institute, 64, Sangmujungang-ro, Seo-gu, Gwangju, 61949 South Korea; 2 Chung-Yeon Korean Medicine Hospital, 64, Sangmujungang-ro, Seo-gu, Gwangju, 61949 South Korea; 3 Department of Acupuncture & Moxibustion Medicine, College of Korean Medicine, Daejeon University, 62, Daehak-ro, Dong-gu, Daejeon, 34520 South Korea; 4 Dongshin Korean Medicine Hospital, 351, Omok-ro, Yangcheon-gu, Seoul, 07999 South Korea; 5 Department of Korean Rehabilitation Medicine, College of Korean Medicine, Daejeon University, 62, Daehak-ro, Dong-gu, Daejeon, 34520 South Korea; 6 Department of Korean Medicine Obstetrics & Gynecology, College of Korean Medicine, Daejeon University, 62, Daehak-ro, Dong-gu, Daejeon, 34520 South Korea; 7 East West Cancer Center, Dunsan Korean Medicine Hospital of Daejeon University, 75, 176 Bun-gil, Daedeok-daero, Seo-gu, Daejeon City, 35235 South Korea; 8 Department of Neuropsychiatry, Dunsan Korean Medicine Hospital of Daejeon University, 75, Daedeok-daero 176 beon-gil, Seo-gu, Daejeon, 35235 South Korea; 9 Department of Internal Medicine, Dunsan Korean Medicine Hospital of Daejeon University, 75, Daedeok-daero 176 beon-gil, Seo-gu, Daejeon, 35235 South Korea

**Keywords:** Knee osteoarthritis, Gyejigachulbutang, Gui Zhi Jia Shu Fu Tang, TJ-18, Keishikajutsubuto, Randomized controlled trial, Protocol, Traditional medicine

## Abstract

**Background:**

Degenerative knee osteoarthritis is a leading cause of disability in the elderly. If patients do not respond to pharmacological or nonpharmacological intervention, total knee replacement surgery is recommended. However, owing to the contraindications and adverse effects of surgery, the need for a new treatment strategy is emerging. Traditional herbal medicine is a widely used intervention in east Asia to treat knee osteoarthritis. Gyejigachulbutang is one of the frequently prescribed herbal formulae. The aim of our study is to evaluate the efficacy and safety of gyejigachulbutang for knee osteoarthritis.

**Methods:**

This study is a randomized, placebo-controlled, patient and assessor blinded, superiority clinical trial. A total of 80 patients with knee osteoarthritis will be enrolled. The participants will be randomly assigned to the gyejigachulbutang or placebo group in a 1:1 ratio in two Korean medical hospitals. Every participant will take gyejigachulbutang or placebo at a dose of 2.5 g three times a day for 4 weeks. Additional follow-up will be conducted 4 weeks after treatment completion. Any concomitant treatment to relive knee pain will not be allowed except for rescue medicine (acetaminophen). The primary outcome will be a comparison of the change in the visual analogue scale score for knee pain from baseline to visit 3 (week 4) for both the treatment and placebo groups. Secondary outcomes include clinical relevance, minimal clinically important difference, disability, quality of life, and safety.

**Discussion:**

This protocol presents a research methodology for clinical trials of gyejigachulbutang for knee osteoarthritis. Various secondary outcomes make this trial more informative. Our trial will provide fundamental evidence for knee osteoarthritis management via herbal medicine treatment.

**Trial registration:**

Clinical Research Information Service (CRIS), KCT0003024. Registered on 25 July 2018.

**Electronic supplementary material:**

The online version of this article (10.1186/s13063-019-3234-6) contains supplementary material, which is available to authorized users.

## Background

Degenerative knee osteoarthritis (KOA) is one of the most common diseases in adults with degenerative changes in the knee joint, such as excessive bone formation and joint deformation [1]. Clinical symptoms of degenerative KOA include mild pain, fatigue, movement disorders, swelling and tenderness, and fricative sounds during exercise. Radiological findings include loss of articular cartilage, structural joint changes, hardening of the sac, and irregularity of the joint surface [2]. Diagnosis of degenerative KOA is based on clinical judgment including past history, physical examination, radiology, and laboratory evaluation. In this process, specific causes such as rheumatoid arthritis, gout, and soft tissue damage should be excluded [3]. In Korea, the prevalence rate of degenerative KOA was 21.1% (19.6–22.8%) in men and 43.8% (42.0–45.6%) in women over the age of 50 years in 2012. The prevalence of KOA increases steadily with age [4]. KOA is a major cause of disability for older patients because progressive loss of articular cartilage leads to joint pain and disability. The quality of life of KOA patients was below the 25th percentile compared with the normative value for healthy people [5].

In terms of a nonpharmaceutical approach to KOA, several interventions such as walking, manual therapy, taping, acupuncture, and thermal agents are recommended. In terms of a pharmaceutical approach, several medications are used ranging from nonsteroidal anti-inflammatory drugs, intra-articular hyaluronate, intra-articular corticosteroids, duloxetine, and short-term weak opioids [6]. If symptoms are severe and the quality of life is low, total knee joint replacement (TKR) is a recommended option for patients who do not respond to conventional nonpharmacologic and pharmacologic treatment [7]. In the US alone, more than 670,000 total knee replacement surgeries were conducted in 2012 [8]. However, there are several adverse events from TKR including venous thromboembolism, joint infection, and myocardial infarction [9]. Total knee replacement is contraindicated in some patients [10]. It is reasonable to assume that the quality of life of the patients who undergo TKR will still be low after surgery [5]. Some patients do not respond to conservative treatment commonly used in clinical practice include medication, physical therapy, injection, massage, exercise, and patient education [11]. Therefore, there is still a need for alternative treatment strategies to reduce the possibility of surgery and to manage pain safely and effectively in KOA patients.

Gyejigachulbutang (GCB; Gui Zhi Jia Shu Fu Tang, Keishikajutsubuto, TJ-18) is a traditional herbal formula widely used in traditional east Asian medicine (TEAM). Gyejigachulbutang is composed of *Cinnamomi Ramulus*, *Paeoniae Radix*, *Atractylodes Lancea Rhizome*, *Zizyphi Fructus*, *Glycyrrhizae Radix*, *Zingiberis Rhizoma*, and *Aconiti Radix Processa*. Historically, GCB has been widely used for several diseases such as influenza, the common cold, arthritis, and muscle pain in clinical practice [12]. Several preclinical and clinical studies on GCB also reported evidence of its usefulness in some diseases such as postherpetic neuralgia [13], chemotherapy-induced neuropathy [14], rheumatoid arthritis [15], neuropathic pain in dental clinics [16], and degenerative osteoarthritis [17]. Despite the wide use of GCB in clinical practice to treat KOA patients, there is inadequate clinical evidence on GCB for KOA treatment.

In terms of clinical benefits, GCB has a pain-relieving effect by warming up the body, improving blood circulation in joints, and lubricating the skin and joint [18]. A case report has described the use of GCB to treat refractory accumulation of synovial fluid in pustulotic arthro-osteitis. GCB reduced the synovial fluid volume, number of neutrophils, and interleukin-8 level in the synovial fluid [19]. In another clinical report, GCB successfully improved arthralgia of somatoform disorder patients who were resistant to painkillers. Therefore, GCB could be an alternative for nonresponders to a painkiller [18]. In another case series, GCB also improved postherpetic neuralgia [20]. This indicates that GCB could be a potential therapeutic option for KOA management [18]. In terms of its mechanism of action, GCB was shown to suppress the levels of proinflammatory mediators such as tumor necrosis factor-α, interleukin-1β, interferon gamma, and interleukin-6 [15]. Nitric oxide (NO) is a highly reactive free radical which mediates acute and chronic inflammation. In the previous study, GCB also suppressed production of inducible nitric oxide synthase (iNOS), which produces NO [15]. Mechanisms of action of the medicinal plants included in GCB have also been investigated. Analgesic effects of Paeoniae Radix and Aconiti Radix Processa are already known [20]. Relieving edema is also an important aim of KOA management [21], and Atractylodes Lancea Rhizome is traditionally known to relieve edema [22]. Several side effects of using conventional drugs for KOA symptoms are already known, such as constipation, excessive sedation, and gastrointestinal complications [23]. According to a previous review, traditional herbal medicine shows less adverse events than does conventional treatment in KOA management, with a better therapeutic effect [21]. As GCB is a traditional herbal medicine characterized as a multi-component multi-target drug, it could have complex therapeutic effects and fewer adverse events compared with a conventional single-compound medication [21]. GCB may be safer and have different indications and mechanism of action compared with conventional treatment. Therefore, we need well-designed clinical trials on the use of GCB in KOA management.

The purpose of this study is to evaluate the efficacy and safety of GCB for degenerative KOA. The primary objective is to assess the efficacy and safety of 4 weeks of GCB treatment compared with a placebo drug for pain reduction measured by a visual analogue scale (VAS) in KOA patients. We will also evaluate the efficacy of GCB in terms of clinical relevance, disability, quality of life, and global assessment in KOA patients.

## Methods/design

### Trial design and study setting

**Fig. 1:**
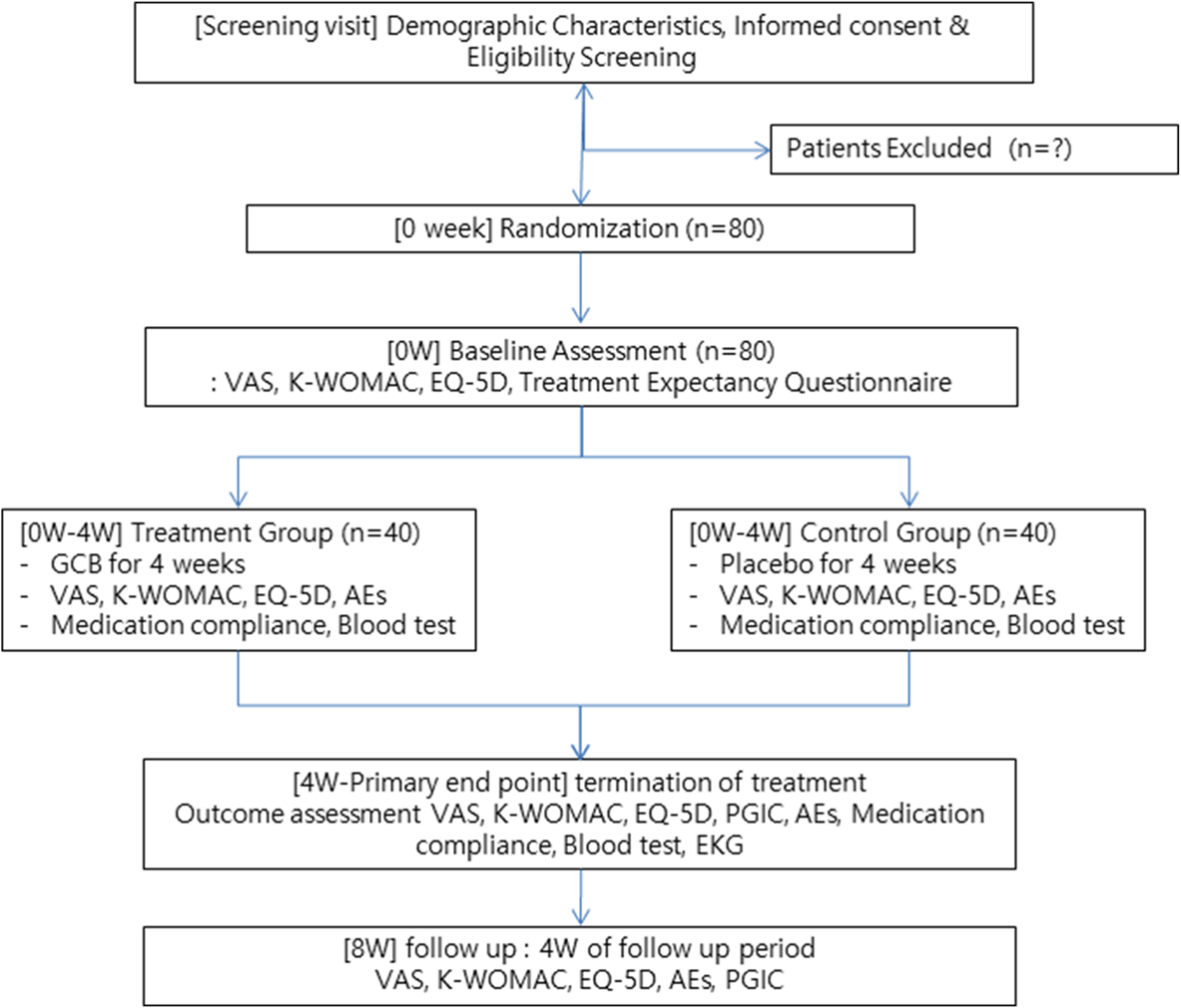
Study flowchart. AE adverse event, EKG electrocardiogram, EQ-5D EuroQoL-5D, GCB gyejigachulbutang, K-WOMAC Korean Western Ontario and McMaster Universities Osteoarthritis Index, PGIC patient global impression of change, VAS visual analogue scale

This study is a randomized, placebo-controlled, patient–physician and assessor blinded, parallel group, two-center, superior clinical trial. Eighty patients who fulfill the inclusion and exclusion criteria will be randomized into GCB and placebo groups in a 1:1 ratio. The study flowchart and trial design are shown in Fig. 1. and Table 1. Recommended items to address in a clinical trial protocol and related documents are described according to the SPIRIT 2013 checklist [24] (Additional file 1).

**Table 1:** Study schedule

Assessment	Enrollment	Treatment phase			Follow-up phase
	Screening (−2 weeks ~ day 0)	Visit 1 (0 weeks)	Visit 2 (2 weeks)	Visit 3 (4 weeks)	Visit 4 (8 weeks)
Informed consent	X				
Inclusion/exclusion criteria	X				
Vital signs and physical examination	X	X	X	X	X
Demographic characteristics	X				
Medical history	X				
Treatment expectancy questionnaire	X				
Blood test^a^	X			X	
Electrocardiogram	X			X	
Urine hCG (only women)	X				
Radiography of both knees	X				
Randomization		X			
VAS	X	X	X	X	X
K-WOMAC		X	X	X	X
EQ-5D		X	X	X	X
PGIC		X	X	X	X
Medication compliance			X	X	
Check rescue medicine and concomitant treatment			X	X	X
Safety assessment		X	X	X	X
Blinding test				X	
Medication administration		X	X		
Participant education	X	X	X	X	

If necessary unscheduled visits are allowed and are recorded in the medical record and case report form

*EQ-5D* EuroQoL-5D, *hCG* human chorionic gonadotrophin, *K-WOMAC* Korean Western Ontario and McMaster Universities Osteoarthritis Index, *PGIC* patient global impression of change, *VAS* visual analogue scale

^a^Blood test consists of red blood cell count (RBC), white blood cell count (WBC), hemoglobin, hematocrit, platelets, erythrocyte sedimentation rate (ESR), C-reactive protein (CRP), aspartate aminotransferase (AST), alanine aminotransferase (ALT), gamma-glutamyl transferase (γ-GTP), total bilirubin, blood urea nitrogen (BUN), creatinine, and electrolytes (Na, K, Cl); rheumatoid factor will be checked only at the screening visit for screening purposes; women of childbearing age are further tested for urine hCG to identify pregnancy before the first treatment; each visit allows a window of 3 days

### Recruitment

Two Korean Medicine Hospitals located in the Republic of Korea, namely Dunsan Korean Medicine Hospital of Daejeon University, Daejeon, Republic of Korea, and Chung-Yeon Korean Medicine Hospital, Gwangju, Republic of Korea, will recruit 56 and 24 outpatients, respectively, in a clinical practice setting. Advertisement of the clinical trial will be posted on the webpage and bulletin board of each hospital, local newspapers, and advertisement boards on public transport systems.

### Eligibility criteria: inclusion criteria

1. Patients over 40 years old.
2. Patients who attained a VAS score of more than 30 mm regarding knee pain during daily life [25].
3. Patients at Grade 2 or higher on the Kellgren-Lawrence Grading Scale [26].
4. Patients who voluntarily decided to participate and signed the written informed consent form after receiving a full explanation of the research objectives and processes.

### Eligibility criteria: exclusion criteria

1. Patients with severe knee trauma in the last 6 months.
2. Patients with a history of knee surgery or planning for surgery within the research period.
3. Patients who received steroid injection treatment within the last 3 months or hyaluronic acid injection treatment within the last 6 months.
4. Patients who received acupuncture, pharmaco-acupuncture or herbal medicine treatment for knee pain relief within the last 1 month.
5. Erythrocyte sedimentation rate (ESR) > 40 mm/h or rheumatoid factor > 20 U/mL on screening examination.
6. Patients with musculoskeletal problems that cause more severe pain in other parts of the body than the knees.
7. Patients who have an uncontrolled heart condition such as angina or congestive heart failure, or liver function abnormality (alanine aminotransferase or aspartate aminotransferase levels 40 IU/L or higher), or kidney function abnormality (creatinine level is outside the range of 0.5–0.9 mg/dL, blood urine nitrate level is outside the range of 6–20 mg/dL), systolic blood pressure greater than 180 mmHg, or diastolic blood pressure greater than 100 mmHg.
8. Patients who are pregnant, nursing, or diagnosed with malignant tumors.
9. Patients who have genetic disorders such as galactose intolerance, or Lapp lactase deficiency, or glucose-galactose malabsorption.
10. Patients who have a significant neuropsychiatric history or who are currently ill from neuropsychiatric disease.
11. Patient who are judged unsuitable for clinical trial participation by the principle investigator.
12. Patient who participated in another clinical trial during the last 3 months.

### Participant withdrawal criteria

1. Violation of inclusion and exclusion criteria.
2. Patients who are unable to continue clinical trial participation due to serious adverse events.
3. Acute systemic reaction (allergy, shock) due to clinical trial drug.
4. The side effects of the rescue medicine (acetaminophen) occur, such as shock (anaphylaxis symptoms), hematologic disorder (thrombocytopenia, granulocytopenia, hemolytic anemia, methemoglobinemia, platelet hypofunction, cyanosis), hypersensitivity (facial swelling, dyspnea, sweating, hypotension, shock), digestive system disorder (nausea, vomiting, poor appetite, gastrointestinal bleeding, digestive ulcer, perforation), skin disorder (rash, allergic reaction, Stevens-Johnson syndrome, Lyell’s syndrome), or other disorders (chronic liver necrosis, acute pancreatitis, chronic hepatitis, kidney toxicity).
5. Patients with systemic diseases that were not found during the screening test.
6. Patients who require surgery or hospitalization due to an accident or other illness.
7. Patients refusing to participate in the clinical trial.
8. Patients who require conventional therapy due to worsening of knee pain.
9. Principal investigator judges that an unavoidable reason to stop participating in the study has occurred for the patient.

### Randomization and allocation concealment

A randomization table for each hospital will be created by an independent statistician with SAS version 9.4 (SAS Institute Inc., Cary, NC). A random number table will be sent to the independent physicians in each hospital who are not engaged in recruitment, assessment, or treatment. Patients will be allocated to the GCB group and placebo group in a 1:1 ratio in each hospital. If the participants voluntarily sign the informed consent and fulfill the eligibility criteria, the clinical research coordinator (CRC) will assign a random number for the participant sequentially in each hospital. The randomization table will be kept in a locked cabinet that only the independent physician can access. The gyejigachulbutang or placebo drug will be packaged on a visit-by-visit basis in advance by the pharmaceutical company according to the randomization table. Clinical trial pharmacists who do not know the allocated group of participants will distribute the packaged drug or placebo according to the randomization number. Gyejigachulbutang and placebo will be made in the same shape and labeled by a pharmaceutical company which is not involved in other processes of the clinical trial. Allocation concealment will be kept throughout the clinical trial.

### Blinding

The CRC, assessor, physician, pharmacist, and patients are blinded to the allocated group, except for the independent statistician who made the random table and the independent physician who has access to the random table in each hospital. If an emergency occurs, the principal investigator requests that the independent physician unblinds the patient. This process will be reported to the institutional review board (IRB). The physician who prescribes the drug and the pharmacist who distributes the drug are also blinded. The labeled clinical trial drug will be offered by the pharmaceutical company. For this process, the random number table will be provided to the company. The assessor who evaluates the clinical trial outcome is also blinded. Unblinding will be done according to the standard operating procedure (SOP) of the Contract Research Organization (CRO). A blinding test will be conducted at visit 3 (week 4) to assess the success of blinding.

### Intervention

#### Study schedule

Table 1 shows the schedule of study. This trial consists of a screening phase, a treatment phase, and a follow-up phase. At the screening visit, each participant will be asked to voluntarily sign a written informed consent form before taking part in the study. At the screening visit, the investigator will conduct demographic surveys, medical examinations (history taking, physical examination, clinical laboratory test, knee x-ray imaging, electrocardiography) and treatment expectancy questionnaire according to the protocol. Participants who fulfill the eligibility criteria should visit again within 2 weeks from the screening visit for visit 1. At visit 1, baseline assessments will be conducted. Participants will be randomized into either the GCB or placebo group at visit 1 (week 0). Participants will take the drug or placebo for 4 weeks. The clinical trial drug will be distributed at visit 1 (week 0) and visit 2 (week 2) by the pharmacist. The treatment phase will be completed at visit 3 (week 4). The primary outcome of the trial will be evaluated without taking rescue medicine on the day of visit 3 (week 4) by blinded assessors. After 4 weeks from visit 3 (week 8), additional follow-up evaluation will be performed at visit 4 (week 8).

#### Intervention protocol

Treatment group participants will receive GCB for 4 weeks. The control group will receive placebo medication for 4 weeks. They will take GCB or placebo granules (2.5 g, three times a day orally) 30 min after every meal. The dose follows the recommended drug dose approval criteria of the Ministry of Food and Drug Safety. Considering a visit window of 3 days, the pharmacist will distribute the trial drug (GCB or placebo) via 51 packs (for 17 days) on visit 1 and visit 2. Participants should return the unused trial drug and compliance will be calculated at visit 2 and visit 3.

#### Clinical trial drug (gyejigachulbutang or placebo)

Gyejigachulbutang (GCB, TJ18) will be manufactured by Tsumura & Co. (Tokyo, Japan). One pack of gyejigachulbutang is composed of Cinnamomi Ramulus 1.33 g, Paeoniae Radix 1.33 g, Atractylodes Lancea Rhizome 1.33 g, Zizyphi Fructus 1.33 g, Glycyrrhizae Radix 0.66 g, Zingiberis Rhizoma 0.33 g, and Aconiti Radix Processa 0.16 g. These raw materials will be extracted and concentrated to 2.5 g per pack.

The placebo drug will be manufactured by Kyungjin Pharmaceutical & Co. (Icheon, Republic of Korea) according to Korean Good Manufacturing Practice standards. The Placebo drug is composed of caramel coloring, lactose, and corn starch. The placebo drug is similar in shape, color, taste, and smell to GCB. Both GCB and placebo drugs will be packaged and labeled by Kyungjin Pharmaceutical & Co. considering the random number table.

#### Concomitant treatment

In principle, all patients are prohibited from using traditional medicine interventions (including acupuncture, moxibustion, herbal medicine, cupping, etc.), conventional medication, injection treatment, surgery, physical therapy, manual therapy, and exercise therapy to improve knee pain. However, concomitant intervention for the treatment of other diseases or adverse events that would not affect the results of this trial are allowed under the judgment of the principle investigator. If a patient’s concomitant treatment is expected to affect the outcome of this trial, the patient will be dropped from the trial.

#### Rescue medication

If the participants are suffering from severe knee pain we allow them to take a rescue medication*.* Acetaminophen (maximum daily dose of 3000 mg or less, six tablets per day) will be provided as a rescue medication and should be taken only when the pain is unbearable. The total amount of rescue medication consumption will be recorded at each visit. Patients will be advised not to take rescue medication to relieve knee pain on the days of visit 2 (2 week), visit 3 (week 4), or visit 4 (week 8). If necessary, we will instruct the participants to take the pain control medication after assessment of the clinical outcome. If a patient’s rescue medication consumption is expected to affect the outcome of this trial, the patient will be dropped from the trial.

### Outcome measures: primary endpoint

As pain is the most common complaint of degenerative arthritis, we selected the VAS as the primary outcome to assess pain severity [27]. The primary outcome of our study is a change in the value of VAS score from baseline (visit 1) to week 4 (visit 3). The VAS score evaluates a person’s pain intensity level. In our trial, the participants are asked to place a mark on a 100 mm horizontal line with the question ‘how much pain did you have during last 3 days’. The beginning of the line illustrates ‘no pain’ and the end of the line indicates the ‘worst imaginable pain’. To extract the outcome value, the investigator measures the distance in millimeters between ‘no pain’ and the marked point by the participant.

### Outcome measures: secondary endpoints

#### Pain

Change in the value of VAS score from baseline (visit 1) to week 2 (visit 2) and from baseline (visit 1) to week 8 (visit 4).

The minimal clinically important difference (MCID) helps interpret the results of clinical trials at the individual level [28]. For the assessment of MCID in the VAS score for knee pain [28, 29], the proportion of participants with a VAS score decrease of > 30% from baseline will be compared between the treatment and control groups at week 4 (visit 3) and week 8 (visit 4). We will also compare the degree of improvement between the mild group (VAS score lower than 50%) versus the severe group (VAS score above 50%) of the participant’s VAS score at week 4 (visit 3) and week 8 (visit 4).

#### Disability

The validated Korean version of the Ontario and McMaster University Osteoarthritis index (K-WOMAC) will be rated to evaluate disability associated with joint pain, stiffness, and functional status in the knees during the last 48 h [30, 31]. The change in the K-WOMAC score at week 2 (visit 2), week 4 (visit 3), and week 8 (visit 4) will be compared between both groups. The K-WOMAC consists of 24 questions (five about pain, two about stiffness, and 17 about physical functions) and can be completed in less than 5 min. A total K-WOMAC score of 96 points and higher means poor status.

#### Quality of life

The three-level version of the Euroqol-5D (EQ-5D-3 L) is a valid and reliable self-reporting questionnaire that measure the patient’s health status for clinical and economic appraisal using a Likert scale and a VAS [32, 33]. The change in the EQ-5D score at week 2 (visit 2), week 4 (visit 3), and week 8 (visit 4) will be compared between the two groups.

#### Global assessment

The patient global impression of change (PGIC) is a valid outcome measure that is based on a seven-point Likert scale. The scale ranges from ‘much better’, ‘better’, ‘somewhat better’, ‘no change’, ‘somewhat worse’, and ‘worse’ to ‘much worse’ [34]. ‘Much better’ is rated as 7 points and ‘much worse’ as 1 point on the PGIC. With this scale, it is possible to dichotomize the participant’s response into two groups, namely those that have ‘improved’ (ratings 5 to 7) and those have ‘not improved’ (ratings 1 to 4). The change in PGIC score and proportion of ‘improved’ patients between the two groups will be compared at week 4 (visit 3) and week 8 (visit 4).

### Safety assessment

In this study, adverse events (AEs) are defined as any undesirable medical findings that occurred after the start of a clinical study regardless of medication. Participants are educated to report any kind of AE during the clinical trial. At every visit, all AEs will be assessed and recorded including vital signs, patient complaints, abnormal laboratory results, and physical examinations. For the safety assessment of GCB, we will conduct a liver function test, renal function test, electrocardiography, and blood cell count at visit 3 (week 4). All identified AEs will be documented in the case report form regardless of their relevance to the medication. Adverse event investigation includes symptoms or diseases, date of occurrence, disappearance date, severity, frequency of occurrence, result of AEs, causality, intervention for AEs, discontinuation of treatment, and unexpectedness. The cause of AEs regarding GCB will be assessed according to the World Health Organization’s Uppsala Monitoring Centre system [35]. When serious adverse events occur, we will withdraw the participants and report to the IRB within 15 working days. The proportion of AEs between both groups will be statistically compared using the chi-square test or Fisher’s exact test.

### Data management and quality control

Monitoring will be conducted by the CRO three times at each hospital for quality control of the data. The monitoring staff will check to ensure processes of the trial were appropriately conducted according to the approved protocol. The first monitoring will occur when the first participant enrolls. The next monitoring will occur when 50% of the participants are recruited. The last monitoring will occur when the last participant is enrolled. To improve data quality, we will use an electronic case report form (e-CRF) system [36]. The e-CRF system will conduct data validation by correcting missing data, range checks, inconsistent data, and deviation from the protocol. An audit is not scheduled in our trial.

### Sample size calculation

The primary outcome is change in the value of the VAS from baseline to visit 3 (week 4) between the two groups. The null hypothesis is there is no mean difference in VAS score change from baseline to visit 3 between the GCB group and the placebo group. There are no previous studies on the effects of GCB on KOA. In previous research on the MCID of absolute VAS score change in KOA patients, MCID of VAS change was 19.9 mm (95% confidence interval 17.9 to 21.6) [28]. The standard deviation (SD) of change in the VAS value was 22 mm. Therefore, we adopted a mean difference of 17.9 mm and SD of 22 mm. The required number of patients for each group is 32 considering a 1:1 allocation ratio, two-tailed superiority test, a test power of 90% (1 – β) with a significance level of 5% (α).

Considering a 20% dropout rate, we calculated the sample size for each group to be 40 patients per group. A total of 80 patients are therefore required in our trial.

### Statistical analysis

Continuous variables will be expressed as mean and standard deviations. If the data are not normally distributed, median and interquartile range will be presented. Categorical variables will be expressed as frequencies and ratios (%). The main analysis of the primary outcome will adopt a full analysis set (FAS) analysis. A per-protocol (PP) set will be used for subanalysis for sensitivity. FAS is defined as the participant taking the clinical trial drug at least once and has undergone primary outcome assessment at least once. If the participants violated the inclusion/exclusion criteria, did not take the clinical trial drug once, or did not undergo outcome assessment then the participants will be excluded from the FAS. The PP set is defined as follows: 1) compliance of medication consumption is over 70%; 2) every outcome was evaluated; and 3) the patient completed the clinical trial without major protocol violation. Missing data will be replaced using the last observation carried forward (LOCF) analysis method. In primary outcome analysis, the change in value of VAS from baseline to visit 3 (week 4) between the two groups will be compared using analysis of the covariance (ANCOVA) method. Baseline VAS scores will be used as covariates, and each group will be a fixed factor. If there is another baseline variable which was statistically different between the two groups, that variable will also be used as a covariate.

In secondary outcome analysis, continuous variables will be compared using the independent *t* test or the Wilcoxon rank-sum test. Categorical variables will be compared using the chi-square test or Fisher’s exact test. For statistical testing of trends of outcome variables over time, repeated measurements of analysis of variance will be used. Dunnett’s correction method will be applied for multiple comparisons in the secondary outcome analysis. If necessary, we will conduct subgroup analysis according to sex, age, duration of disease, expectancy of treatment, or pain severity. The level of significance will be 5% (two-tailed test). Data will be analyzed with SAS version 9.4 (SAS Institute Inc., Cary, NC). Interim analysis is not planned.

### Ethics approval and registration

The IRBs of Dunsan Korean Medicine Hospital of Daejeon University (IRB approval no. DJDSKH-18-DR-10) and Chung-Yeon Korean Medicine Hospital (IRB approval no. CYIRB-2018-04-002) have approved the protocol. We registered our clinical trial protocol on the Clinical Research Information Service (CRIS), which is one of the primary registries of the World Health Organization International Clinical Trials Registry Platform (CRIS no. KCT0003024; https://cris.nih.go.kr/cris/search/search_result_st01.jsp?seq=11667/).

## Discussion

In our randomized clinical trial, we adopted a placebo-controlled design to enhance the internal validity of the trial. We tried to assess not only pain severity but also clinical relevance, physical function, quality of life, and safety. Clinical relevance is important in musculoskeletal disease. It is already known that comparing the proportion of predefined responders is much more informative than VAS pain score reduction in placebo-controlled trials, especially when the VAS score is not normally distributed [37, 38]. Our study will provide important additional information about GCB treatment. Regular monitoring will enhance the quality of our study data. However, a relatively short treatment duration and follow-up period is a limitation of our study. A subjective primary outcome is another limitation. Even though we will make our placebo drug as similar as possible to GCB, we might not reproduce the unique smell and taste of GCB, which may affect the result of the research. We will assess blindness at visit 3 by a blind assessment questionnaire. We expect our trial result will provide preliminary evidence about the efficacy of GCB for KOA treatment. It will be useful for researchers, physicians, stakeholders, and patients.

## Trial status

The final protocol version is 2.0 and dated 26 July 2018. This trial is currently recruiting participants. Recruitment began on 1 September 2018. We expect the recruitment phase to be complete by May 2019.

## Additional file

Additional file 1: SPIRIT 2013 checklist: recommended items to address in a clinical trial protocol and related documents. (DOC 121 kb)

## Abbreviations

AE: Adverse event
CRC: Clinical research coordinator
CRIS: Clinical Research Information Service
CRO: Contract Research Organization
e-CRF: Electronic case report form
EQ-5D: Euroqol-5D
ESR: Erythrocyte sedimentation rate
FAS: Full analysis set
GCB: Gyejigachulbutang
IRB: Institutional review board
KOA: Knee osteoarthritis
K-WOMAC: Korean version of the Ontario and McMaster University Osteoarthritis index
MCID: Minimal clinically important difference
PGIC: Patient global impression of change
PP: Per-protocol
SD: Standard deviation
SOP: Standard operating procedure
TEAM: Traditional east Asia medicine
TKR: Total knee joint replacement
VAS: Visual analogue scale
